# Synthetic microbiology as a source of new enterprises and job creation: a Mediterranean perspective

**DOI:** 10.1111/1751-7915.13326

**Published:** 2018-10-16

**Authors:** Cristina Vilanova, Manuel Porcar

**Affiliations:** ^1^ Darwin Bioprospecting Excellence SL. Paterna Spain; ^2^ I2SysBio (Institute for Integrative Systems Biology) Universitat de València‐CSIC Paterna Spain

The European biotech industry is proving to be robust against the challenges arising during the last years. Despite geopolitical complexity, regulatory uncertainty and the growing potential of the Asian bio‐based industry, European biotechs are still growing in number and revenues, and even SMEs have been able to ‘stay in the course’ by developing new business models and keeping R&D as a pillar of competitiveness. The UK and Germany host the most powerful hubs of biotech companies. In 2017, nearly 30% of all of Europe's venture capital went to UK‐based biotechs, whereas the German biotech hub led the league in terms of a number of dedicated biotechnology firms, with more than 500 SMEs (Biotechnology Report 2017). Roughly, half of the European biotech companies are settled in these major hubs, with Switzerland and Sweden being also remarkable hotspots for the biotechnology‐based industry.

Although it is yet to be improved, the scenario in Southern Europe is far from discouraging. French companies such as GenSight and Pharnext (both developing innovative treatments for ophthalmic and neurology diseases) are flagship examples of top‐ranking European biotechs in terms of capitalization and revenues (Van Beneden, 2018). Beyond particular cases, biotechnology hubs are quickly developing in France, Italy and Spain (Fig. 1A). In fact, more than 120 companies are settled in Madrid, 141 in Lombardy and more than 150 in the biotechnology hubs of Paris and Barcelona. While Northern and Central Europe biotechs largely focus on drug discovery and manufacturing of biomaterials, there is a growing number of Southern Europe companies with business models centred in the development of new products for the agro‐food sector, and in the so‐called ‘platform technologies’ (i.e. DNA sequencing, multiomics or bioinformatic data mining; Allansdottir *et al*., 2002). The rise of industrial biotechnology in Southern Europe is also measurable by the increasing number of R+D projects and funds granted by the EC throughout the last decade (Fig. 1B, C respectively).

**Figure 1:**
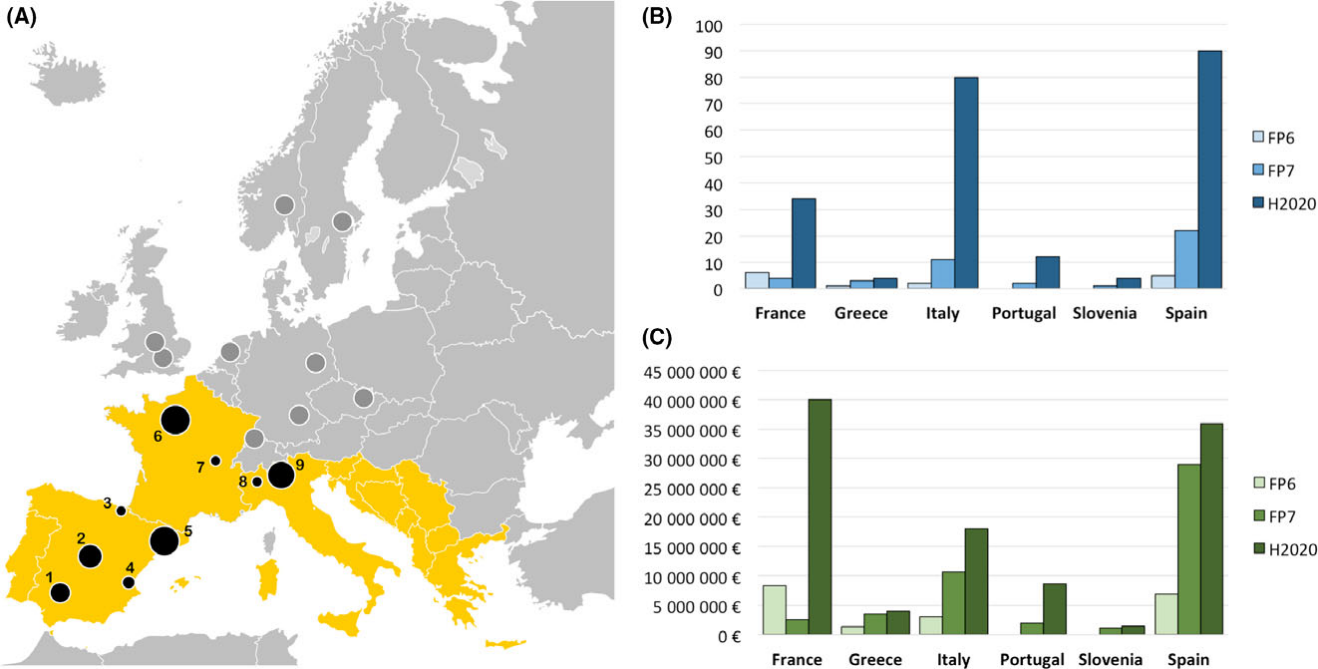
(A) Distribution of European biotech hubs (> 50 companies) with especial emphasis on Southern Europe. The size of the black spots (hubs) is proportional to the number of biotech companies conforming the hub (1: Sevilla; 2: Madrid; 3: Basque Country; 4: Valencia; 5: Barcelona; 6: Paris; 7: Lyon; 8: Piedmont and 9: Lombardy); (B) Evolution of the number of R+D biotech projects funded by the SME program of the EC throughout the FP6, FP7 and H2020 (estimated) periods; (C) Evolution of EC funding received by Southern Europe biotechs throughout the FP6, FP7 and H2020 (estimated) periods. Data retrieved from the CORDIS database.

This growth is connected with the conceptual and technical progresses that have taken place in biological engineering in the last years. Indeed, it would be wrong to merely link the success of biotechnological companies with the public and private funds gathered, particularly in the case of synthetic biology. In fact, the standardization of biotechnology is one of the main goals of synthetic biology, which aims at the design of biological systems from an engineering perspective (Khalil and Collins, 2010). If achieved, biological standardization is expected to boost the development of biotechnological products and processes, by improving the predictability and robustness of their underlying biological circuits. However, there are important cultural and technical issues that certainly hamper the development of synthetic biology in Europe. One of those is a lack of trust in the analogy between biology and engineering that lies at the very core of synthetic biology. Indeed, the field has classically identified cells as living machines, but there are solid reasons to cast doubts on the exactness of such a metaphor (Porcar and Peretó, 2016). It has to be stressed that successes in most of the biotechnological projects considered as synthetic biology have in fact been the consequence of trial‐and‐error strategies rather than the result of a generalized standardization in biology. The difficulties biological standardization faces are the intraspecific variation in heterologous protein production (i.e. variation depending on the strain of *E. coli* being transformed); the cell‐to‐cell variation in output signal intensity or the non‐orthogonal effects of simple biological circuits on each other, as we have previously reported (Vilanova *et al*., 2015). All these limitations can be summarized in a simple way: true standards in synthetic microbiology do not exist to this date.

Both the potential and limitations of standardization in SB are exemplified by the international Genetically Engineered Machine (iGEM) competition, in which students worldwide present synthetic biology projects based on organisms engineered from a toolbox of BioBricks™. However, Biobricks™ has limitations as universal building blocks in SB (Vilanova and Porcar, 2014; Valverde *et al*., 2016). Interestingly, these limitations have not been an obstacle for a dense network of synthetic biology enterprises to flourish in the US, while in Europe, the landscape of synthetic biology enterprises is sparse by comparison, even if the main efforts to overcome a paucity of standardization are totally or partially of European origin, such as the development of a Standard European Vector Architecture (SEVA; Silva‐Rocha *et al*., 2013), or the standardized representation of SB designs known as the Synthetic Biology Open Language (SBOL; Galdzicki *et al*., 2014). A clear definition of the notion of standard in biology, with all its limitations and possibilities, is still thus much needed, and it would certainly contribute towards a richer landscape of synthetic biology enterprises, particularly in Southern Europe. A new paradigm of biological standardization is thus central for synthetic biology in Europe to consolidate.

In this scenario, one of us (MP) is coordinating the EU‐funded CSA on synthetic biology BIOROBOOST, which has as the main challenge to translate a core concept for engineering disciplines – standardization – into the biological realm. The proposal encompasses research groups and institutions worldwide as well as enterprises (two of which from Southern Europe) working on metrology, cloning techniques, metabolic engineering, genome reduction, etc. BIOROBOOST aims at further developing standards in biology in a holistic, systematic way, from the biological parts to the human procedures and techniques. The project will also define a limited set of specific chasses, adapted to particular industrial and ecological niches, such as thermophilic or halophilic environments, and will gain insight into cultural (lab‐to‐lab procedure variations) aspects. Indeed, standardization is not only about biological parts, but also about the way techniques, protocols and even companies’ ‘cultures’ are developed. The ‘cultural Achilles heel’ of standardization in biology has been very poorly explored to date, but is brightly exemplified by Nobel Prize laureate Murray Gell‐Mann, who stated that ‘a scientist would rather use someone else's toothbrush than another scientist's nomenclature’. This failing must be rectified as soon as possible since reluctance to accept standards is a veritable obstacle for their implementation.

In summary, Mediterranean Europe holds great promise as a new pole for synthetic biology, but this potential will only be met provided that the necessary conditions for it to flourish are present. The main condition is continued public and private support for enterprises and academia involved in central issues, such as biological standardization, that will determine the fate of synthetic biology.

## Conflict of interest

None declared.
